# Greater In-Hospital Care and Early Rehabilitation Needs in People with COVID-19 Compared with Those without COVID-19

**DOI:** 10.3390/jcm11133602

**Published:** 2022-06-22

**Authors:** Kristen Grove, Dale W. Edgar, HuiJun Chih, Meg Harrold, Varsha Natarajan, Sheeraz Mohd, Elizabeth Hurn, Vinicius Cavalheri

**Affiliations:** 1 Department of Physiotherapy, Royal Perth Hospital, Royal Perth Bentley Group, East Metropolitan Health Service, Perth, WA 6000, Australia; kristen.grove@health.wa.gov.au (K.G.); m.harrold@curtin.edu.au (M.H.); 2 Department of Physiotherapy, Fiona Stanley Hospital, South Metropolitan Health Service, Murdoch, WA 6150, Australia; dale.edgar@health.wa.gov.au (D.W.E.); varsha.natarajan@health.wa.gov.au (V.N.); 3 Burn Injury Research Node, University of Notre Dame Australia, Fremantle, WA 6160, Australia; 4 Division of Surgery, Medical School, University of Western Australia, Crawley, WA 6009, Australia; 5 Fiona Wood Foundation, Fiona Stanley Hospital, South Metropolitan Health Service, Murdoch, WA 6150, Australia; 6 Curtin School of Population Health, Curtin University, Bentley, WA 6102, Australia; h.chih@curtin.edu.au; 7 Western Australian Health Translation Network, Perth, WA 6009, Australia; 8 Curtin School of Allied Health, Curtin University, Bentley, WA 6102, Australia; 9 Department of Cardiology, Fiona Stanley Hospital, South Metropolitan Health Service, Murdoch, WA 6150, Australia; mohd.sheeraz@health.wa.gov.au; 10 Department of Physiotherapy, Sir Charles Gairdner Hospital, North Metropolitan Health Service, Nedlands, WA 6009, Australia; elizabeth.hurn@health.wa.gov.au; 11 Allied Health, South Metropolitan Health Service, Murdoch, WA 6150, Australia

**Keywords:** COVID-19, rehabilitation, respiratory illness, disease severity

## Abstract

↔This study aims to compare the characteristics, in-hospital data and rehabilitation needs between those who tested positive versus negative for COVID-19 during hospitalisation with suspected COVID-19. In this cross-sectional study, a convenience sample of adults admitted to Western Australian tertiary hospitals with suspected COVID-19 was recruited. Participants were grouped according to their polymerase chain reaction (PCR) test result into COVID-19 positive (COVID+) and COVID-19 negative (COVID−) groups. Between-group comparisons of characteristics of the participants and hospital admission data were performed. Sixty-five participants were included (38 COVID+ and 27 COVID−; 36 females [55%]). Participants in the COVID+ group had greater acute hospital length of stay (LOS) (median [25–75th percentile] 10 [5–21] vs. 3 [2–5] days; *p* < 0.05] and only those with COVID+ required mechanical ventilation (8 [21%] participants). Twenty-one percent of the COVID+ participants were discharged to inpatient rehabilitation (7% of the COVID− participants). Of note, pre-existing pulmonary disease was more prevalent in the COVID− group (59% vs. 13%; *p* < 0.05). Within the COVID+ group, when compared to participants discharged home, those who required inpatient rehabilitation had worse peripheral oxygen saturation (SpO_2_) on admission (86 ± 5.7% vs. 93 ± 3.8%; *p* < 0.05) and longer median LOS (30 [23–37] vs. 7 [4–13] days; *p* < 0.05). Despite having less people with pre-existing pulmonary disease, the COVID+ group required more care and rehabilitation than the COVID− group. In the COVID+ group, SpO_2_ on hospital presentation was associated with LOS, critical care needs, mechanical ventilation duration and the need for inpatient rehabilitation.

## 1. Introduction

Throughout the COVID-19 pandemic, emergency departments (ED) were filled with people being treated for COVID-19. A challenge created by COVID-19 is that no specific clinical features discern the disease from other respiratory illnesses [1]. Common methods to quantify respiratory compromise assist clinicians in planning and delivering appropriate care for people presenting with respiratory symptoms [2,3,4,5]. It is well-known, in those who are hospitalised with illness causing respiratory compromise, that the duration of the immobility and frequency of mobility during hospitalisation impact functional outcomes, exercise capacity and discharge destination [6,7,8,9,10,11,12,13]. It is also well-known that critical illness with prolonged mechanical ventilation may result in long-lasting functional impairments attributed to intensive care unit (ICU)-acquired weakness (ICU-AW) [14,15].

Approximately 15% of symptomatic COVID-19 cases develop severe disease requiring oxygen support [16]. Simple bedside markers of respiratory compromise in COVID-19 can predict critical care needs and mortality [17,18,19]. Those hospitalised with severe COVID-19 often have a prolonged length of stay (LOS) and may require long periods of mechanical ventilation; thus, they are at high risk for developing ICU-AW [20,21,22,23]. Importantly, people hospitalised with COVID-19 have demonstrated persistant functional impairment requiring inpatient and/or outpatient rehabilitation [23,24,25]. A recently published study demonstrated that new disability attributed to COVID-19 in mechanically ventilated patients with respiratory failure occurs at the same rate as those ventilated for other causes of respiratory failure [26]. However, the burden of COVID-19 during emergency department presentation and hospital admission, when compared to the burden of other illnesses that lead to respiratory symptoms, is yet to be investigated.

The primary aim of this study was, for adults who presented with suspected COVID-19 in EDs, to compare characteristics and in-hospital data between those who tested positive versus negative for COVID-19. The secondary aim was, in those who tested positive for COVID-19, to explore factors linked to discharge destination. In this group, we also provided details of the assessment of physical function.

## 2. Materials and Methods

### 2.1. Study Design and Participants

This report presents the combined data and outcomes of the hospitalised cohort recruited in the Life AfTER COVID-19 (LATER-19) trial [27,28] and acute care data shared through sister-trial provisions with the Western Australia (WA) node of the International Severe Acute Respiratory International Consortium (ISARIC) Trial, established in March 2020 (REG 0000003976). Ethics approval for the LATER-19 trial was granted by the South Metropolitan Health Service (SMHS) Human Research Ethics Committee (HREC) with reciprocal approvals from North and East Metropolitan Health Services (NMHS, EMHS) (REG 0000004040). Data were collected between June 2020 and October 2021. Inclusion criteria were: (i) people ≥18 years; (ii) admitted to three Western Australian hospitals with suspected COVID-19. Participants were excluded if: (i) they had a cognitive or communication impairment thought to limit their ability to complete the self-report measures; (ii) or a neuromuscular impairment limiting ability to complete the physical function test, or (iii) known mental illness. Due to low case numbers in Western Australia in 2020, the protocol was amended to allow retrospective recruitment. All participants provided informed written consent. The trial was registered by Australian and New Zealand Clinical Trial Registration (ANZCTR): ACTRN12621001067864.

### 2.2. Participants Who Tested Positive and Negative for COVID-19

A convenience sample of consecutive patients admitted to respiratory wards was used. As per policy of the time, all those presenting to hospital with symptoms of respiratory illness took a PCR test. Participants who were confirmed to have a positive result by laboratory PCR test formed the COVID+ group. Those who returned a negative result formed the COVID− group.

### 2.3. Data Collection and Outcomes

Under sister trial provisions, admission and emergency department (ED) data from participants were captured when recruited to the WA node of the ISARIC Trial. Data collected during the LATER-19 study were extracted as per protocol [27] and linked to the first contact information.

### 2.4. Data Collected in Both Groups

The variables included in this study comprised: (i) those collected at ED presentation (comorbidities; body mass index (BMI)); markers of respiratory compromise in ED (i.e., peripheral oxygen saturation (SpO_2_) on room air, oxygen flow rate (L/min) and device required to stabilise SpO_2_); (ii) factors during admission (highest oxygen flow rate and device, mobility status, length of hospital stay to treat the acute condition (LOS), ICU admission, mechanical ventilation, days ventilated, discharge destination); and (iii) critical care needs, defined as the need for any of the following during admission: low-flow O_2_ therapy ≥10 L/min, high-flow O_2_ therapy, non-invasive ventilation (NIV) or mechanical ventilation [17]. Mobility status during admission was quantified by applying the ICU Mobility Scale (IMS) [29,30] to retrospectively collect mobility data from the patient medical record, recorded as a snapshot lowest IMS score on a single day during admission. The score ranges between 0 and 10, where <2 infers ‘Rest in Bed’ (RIB), and a score >8 infers independent ambulation. When an IMS score of <2 was recorded, number of days of RIB was also recorded. Inpatient rehabilitation was defined as commencing when: a participant was discharged to a rehabilitation facility; or transferred within hospital to a rehabilitation ward; or if documented within the medical record that the participant remained as an inpatient due to rehabilitation needs.

### 2.5. Data Collected in the COVID+ Group Only

Those who were prospectively recruited within the COVID+ group completed an assessment of physical function during admission via the 1STS [31]. Assessments were planned to be performed on admission and then every second day following admission, with continuous SpO_2_ monitoring. The level of assistance required to complete one repetition of the 1STS was standardised by scoring the STS portion of the Chelsea Critical Care Physical Assessment Tool (CPAx) [32,33]. Assessment of the 1STS did not proceed if the participant had low-flow oxygen requirements >4 L/min, raised troponin, resting HR > 120 bpm, abnormal/unstable baseline heart rhythm, delirium, postural hypotension, STS CPAx score <4 during ICU admission, or participant declined. Assessment was stopped if HR response was of concern (i.e., >HRmax of 220-age; or beta blocker resting HR + 40); reported new chest pain or dizziness; or SpO_2_ fell to <88% during the test (could re-commence within the minute when SpO_2_ ≥ 88%).

### 2.6. Statistical Methods

Characteristics of the participants were described using frequency (percentage), mean ± standard deviation, or median [25th to 75th percentile]. Comparison between participants in the COVID+ and COVID− group (group comparison 1) as well the comparison of COVID+ participants according to their discharge destination (i.e., inpatient rehabilitation versus discharged home; group comparison 2) were performed using Chi-squared tests, independent samples *t*-tests or Mann–Whitney U tests. Patient characteristics were also reported in percentage by length of stay, critical care, mechanical ventilation and rehabilitation status among participants in the COVID+ group. All analyses were performed using Stata IC/14.1 (Stata Corp, College Station, TX, USA).

## 3. Results

### 3.1. Participants

The results of the recruitment process for the LATER-19 trial are described elsewhere [28]. A total of 65 hospitalised participants were included in the analyses for this report. Of these, 38 participants tested positive for COVID-19 and formed the COVID+ group. Sixteen of the 38 participants (42%) in the COVID+ group were prospectively recruited and 22 (58%) were retrospectively recruited. The 27 participants who tested negative for COVID-19 and formed the COVID− group of the study were retrospectively recruited.

### 3.2. Comparison of Participants in the COVID+ versus COVID- Group

Participants in the COVID+ (n = 38) and COVID− (n = 27) group were balanced for sex, age and BMI (Table 1). The COVID− group presented a higher prevalence of pre-existing respiratory disease than those in the COVID+ group (59% vs. 13%; *p* < 0.05). In the COVID+ group, there was a trend towards a greater critical care need, and participants were more likely to be admitted to an ICU and receive mechanical ventilation (Table 1). Ten participants in the COVID+ group (28.6%) scored IMS < 2 during admission compared to 0 (zero) participants in the COVID− group (*p* < 0.05). The median LOS-acute of those in the COVID+ group was a week longer than those in the COVID− group (*p* < 0.05).

### 3.3. Comparison of COVID+ Participants Grouped According to Their Discharge Destination

The comparison of COVID+ participants grouped according to their discharge destination is presented in Table 2. Eight (21%) participants in the COVID+ group were discharged to inpatient rehabilitation, and thirty (79%) were discharged home. The mean LOS-rehabilitation of the eight participants discharged to inpatient rehabilitation was 16.3 days (95% CI 9.3 to 23.2). Pre-admission characteristics were similar between the groups. When compared to participants discharged home, those who required inpatient rehabilitation had worse SpO_2_ on admission (mean difference: −6.9, 95% CI of mean difference: −10.5, −3.2; *p* < 0.05) and longer median LOS (30 [23 to 37] vs. 7 [4 to 13] days; *p* < 0.05).

Factors of respiratory compromise on ED presentation such as SpO_2_ on room air and oxygen flow required to stabilise, appear to differ by LOS-acute and critical care needs (Table 3). In addition to respiratory compromise, a large percentage of patients needing mechanical ventilation (63%) and a long period of RIB over 9 days (83%) required rehabilitation. Those who maintained independent mobility (IMS > 8) did not require rehabilitation.

### 3.4. Data Collected in the COVID+ Group Only (1STS)

Of the 38 participants in the COVID+ group, 12 (32%) had the opportunity to complete repeated 1STS during acute hospital admission. The characteristics of this subgroup of participants are described in Table 4. All of these participants were located at one site. The 1STS was completed on 29 occasions with 7 participants completing multiple attempts. For those who had the opportunity to participate in 1STS, reasons for assessments not proceeding included: weakness, deafness, delirium, postural hypotension, high oxygen requirement or participant declined. There were no occasions of adverse events related to undertaking the 1STS in an inpatient setting.

## 4. Discussion

In this study of patients hospitalised with suspected COVID-19, those with COVID+ and COVID− demonstrated similar baseline characteristics and respiratory compromise at ED presentation. Although those who were COVID− had higher rates of pre-existing pulmonary disease, those who were COVID+ demonstrated higher care needs during admission via higher rates of ICU admission, mechanical ventilation, RIB (IMS < 2), and LOS-acute. It is notable that, when comparing critical care needs rather than ICU admission, although the rate of critical needs within the COVID+ group remains higher, the difference between groups is less. This difference in results may acknowledge the presence of instances of pre-existing clinical frailty barring admission to an ICU, as well as accounting for those prophylactically admitted to an ICU for monitoring. Both groups were admitted under the same jurisdictional conditions early in the pandemic in a health care system that was not overwhelmed [34]. This result suggests that COVID-19 initiates a greater acute burden of care than other illnesses with a similar level of initial respiratory compromise. This is perhaps in contrast to Hodgson’s finding of comparable rates of new disability in both COVID+ and COVID− at 6 months post mechanical ventilation for acute respiratory failure [26].

For those with COVID+, the results for the impact of respiratory compromise at the point of admission on critical care needs, including mechanical ventilation, echo those of much larger studies [17,18]. Specifically, those presenting to an ED with SpO_2_ < 90% on RA, requiring oxygen > 3 L/min to stabilise, were more likely to need mechanical ventilation and/or inpatient rehabilitation, whereas factors of age, comorbidity or BMI had less impact on care trajectory. The duration of mechanical ventilation correlated with a need for inpatient rehabilitation, in keeping with the known impact of ICU-AW, irrespective of the effects of COVID-19 [14,26]. This association complements the impact of mechanical ventilation duration on muscle weakness, the rate of mobility progression and new disabilities in COVID-19 reported elsewhere [25,35,36]. In the absence of mechanical ventilation most participants maintained an independent mobility despite a continuum of initial respiratory compromise. This finding suggests existing clinical practice supporting the early mobilisation in a healthcare system that was not overwhelmed [34]. The characteristics of those with COVID+, requiring rehabilitation in the absence of critical care needs, were investigated, demonstrating the effect of age and pre-admission function on rehabilitation needs. However, this did not specifically address the effect of mobility with acute hospitalisation on need for rehabilitation, nor did it provide a comparison group [37].

This study demonstrates a successful completion of the 1STS without adverse events by those with COVID+ in an inpatient setting. Those completing the 1STS appear to have a greater disease burden than the larger COVID+ group; however, this comparison could not be made formally due to the small group size. This success does offer some value for assessing the response to exercise in the acute period. Given that the frequency of physiotherapy intervention during COVID-19 illness can improve the likelihood of discharge home [38], and the well-established positive effect of early mobilisation in other respiratory and critical care admissions [9,10,11,12,13,14,15], we may consider that the regular completion of 1STS added to the volume of mobility completed by participants who were otherwise bound within their room due to infection control requirements and may have impacted the trajectory of rehabilitation needs. The successful application of the 1STS to safely engage in exercise during acute COVID-19 hospitalisation provides a basis that future larger trials may build from.

### Strengths and Limitations

To our knowledge, this is the first study to provide a comparison group for exploring the trajectory of critical care and rehabilitation needs for COVID-19 patients. To our knowledge, it is also the first study to assess the response to exercise within acute hospitalisation with COVID-19 using repeated measures of a standardised exercise test (1STS).

Low case numbers limited the intended prospective analysis and generalisability of results regardless of the PCR results. Low case numbers also impacted the capacity of matching sub-cohorts by comorbidities and the depth of subsequent between groups comparisons. For instance, exploring types of comorbidities (e.g., heart failure, chronic pulmonary disease) and association with dyspnoea would clarify the needs of COVID+ patients. Future studies would also benefit by controlling for prevalence of comorbidities, such as cardiac and chronic pulmonary disease, to clarify the care needs specifically related to COVID-19. Including a marker of clinical frailty could assist in establishing a functional baseline. The analysis of early mobilisation could be improved by quantifying mobility over repeated measures in conjunction with movement accelerometers to gather more comprehensive movement data.

## 5. Conclusions

Those with COVID+ required more care and rehabilitation than the COVID− group, despite lower rates of a pre-existing pulmonary disease. In the COVID+ group, SpO_2_ on hospital presentation was associated with LOS, critical care needs, mechanical ventilation duration and the need for inpatient rehabilitation. Given the well-established lifetime burden of care required by those with chronic pulmonary disease, this study provides a base that future larger studies may draw from in assessing long-term burden of care in COVID-19.

The 1STS can be safely performed as repeated exercise during acute COVID-19 illness with established physiological parameters. This result could benefit from applications in a larger population to more thoroughly explore responses to exercise and the effect of early intervention during acute COVID-19.

## Figures and Tables

**Table 1:** Characteristics * of the participants grouped according to COVID-19 PCR status.

	COVID− (n = 27)	COVID+ (n = 38)	*p*-Value ^#^
Sex at birth, female	15 (55.6)	21 (55.3)	0.981
Age, yr	67.7 ± 13.9	66.1 ± 11.4	0.601
BMI, kg/m^2^	28.4 ± 7.0	29.9 ± 6.0	0.419
Hypertension, n	10 (37)	14 (37)	0.987
Diabetes, n	5 (19)	7 (18)	0.992
Pulmonary disease ^, n	16 (59)	5 (13)	<0.001
Number of comorbidities (>1)	12 (44)	14 (39)	0.658
SpO_2_ on room air in ED, %	94 [89 to 95]	93 [90 to 95]	0.334
O_2_ flow to stabilise in ED, L/min	0.5 [0 to 3]	0 [0 to 2]	0.398
Critical care needs, n	3 (11)	12 (32)	0.054
Admitted to ICU, n	0 (0)	13 (34)	0.001
Mechanical ventilation, n	0 (0)	8 (21)	0.017
Duration of ventilation (days)	-	12 [8 to 20] **	N/A
LOS-ICU, days	-	14 [4 to 21]	N/A
IMS (score out of 10)	10 [10 to 10]	10 [1 to 10]	0.079
Frequency IMS < 2, n	0 (0)	10 (29)	0.003
Rest in bed, days	-	0 [0 to 2]	N/A
LOS, days	3 [2 to 5]	10 [5 to 21]	0.001
Discharge to inpatient rehabilitation, n	2 (7)	8 (21)	0.175

Abbreviations: Body mass index (BMI), emergency department (ED), oxygen (O_2_), intensive care unit (ICU), ICU Mobility Scale (IMS), length of stay (LOS), peripheral oxygen saturation (SpO_2_), not applicable (N/A; statistical analysis not performed due to one group having no data for such outcome). Note: * Data are frequency (percentage), mean ± standard deviation, or median [25th to 75th percentile]; ** n = 8; ^ pulmonary disease includes chronic pulmonary disease and/or asthma; ^#^: *p*-values were derived from independent samples *t*-tests or Mann–Whitney *U* tests.

**Table 2:** Comparison of COVID+ participants * grouped according to their discharge destination.

	Inpatient Rehab (n = 8)	Discharged Home (n = 30)	*p*-Value ^#^
Sex at birth, female, n	4 (50)	17 (57)	0.736
Age, yr	71 [60 to 76]	68 [61 to 73]	0.691
BMI, kg/m^2^	31.0 ± 9.2	29.7 ± 5.4	0.648
SpO_2_ on room air in ED, %	86.4 ± 5.7	93.3 ± 3.8	<0.001
O_2_ flow to stabilise in ED, L/min	6 [3 to 10]	0 [0 to 1]	0.178
Duration of ventilation, days (n *=* 5)	21 ± 12	6 ± 4	0.091
LOS-ICU, days	23 [21 to 23]	5 [3 to 14]	0.173
IMS (score out of 10)	0 [0 to 1]	10 [10 to 10]	0.100
Rest in bed, days	14 [3 to 17]	0 [0 to 0]	0.080
LOS, days	30 [23 to 37]	7 [4 to 13]	0.005
LOS-rehabilitation, days	16.3 ± 8.3	-	N/A

Abbreviations: Body mass index (BMI), emergency department (ED), intensive care unit (ICU), ICU Mobility Scale (IMS), length of stay (LOS), peripheral oxygen saturation (SpO_2_) not applicable (N/A; statistical analysis not performed due to one group having no data for such outcome). Note: * Data are frequency (percentage), mean ± standard deviation, or median [25th to 75th percentile]; ^#^: *p*-values were derived from independent samples *t*-tests or Mann–Whitney *U* tests.

**Table 3:** Characteristics * of COVID+ patient by length of stay, critical care, mechanical ventilation and rehabilitation.

Colour scale %	FACTORS on ADMISSION	LOS >6	LOS > 9	LOS > 13	Critical Care	Mech Vent	Inpatient Rehab	FACTORS during ADMISSION	Inpatient Rehab
	Admitted to WA Hospital	63	53	40	32	21	21	Admitted to WA Hospital	21
0	FiO_2_ ≥ 0.32 (3 L) to stabilise SpO_2_ in ED	100	100	100	88	75	75	IMS > 8	0
10	SpO_2_ < 90% on RA in ED	100	100	100	88	63	75	>9 days mech vent	100
20	SpO_2_ < 93% on RA in ED	81	69	63	50	38	38	>9 days RIB	83
30	Hypertension	71	64	57	36	21	21	>4 days mech vent or RIB	71
40	Age > 65 years	72	60	44	32	16	20	IMS < 2	70
50	Female	62	52	43	24	19	14	mechanical ventilation	63
60	Sum of comorbidities (total > 1)	67	53	40	20	13	27	IMS < 9	57
70	BMI > 30 kg/m^2^	62	54	39	39	15	23	LOS > 13	53
80	Diabetes	50	50	50	38	38	50	Critical care requirements	50
90	BMI > 35 kg/m^2^	50	50	50	50	33	50	LOS > 9	40
100	Pre-existing pulmonary disease	40	20	20	0	0	0	ICU admission	39

Abbreviations: Body mass index (BMI), emergency department (ED), fraction of inspired oxygen (FiO_2_), intensive care unit (ICU), ICU Mobility Scale (IMS), length of stay (LOS), mechanical ventilation (Mech Vent), peripheral oxygen saturation (SpO_2_), rehabilitation (rehab), rest in bed (RIB), room air (RA), Western Australia (WA). Note: * all values expressed as percentage (%).

**Table 4:** Characteristics * of the participants in the COVID+ group who completed repeated 1 min sit to stand (1STS) during acute hospital admission (n = 12).

Variable	Value
Sex at birth, female, n	6 (50)
Age, yr	70 [65 to 73]
BMI, kg/m^2^	32.6 [27.3 to 34.8]
SpO_2_ on room air in ED, %	90.5 [84.5 to 92.0]
Critical care needs, n	5 (42)
ICU admission, n	4 (33)
Mechanical ventilation, n	3 (25)
LOS-ICU, days	17.5 [10 to 31]
LOS, days	19 [10 to 29]
Discharge to inpatient rehabilitation, n	5 (42)
1STS commenced, days post PCR	14 [11 to 20]
1STS occasions, n	29
STS repetitions	16 [12 to 22]
In test O_2_ Flow, L/min	1.0 [0.0 to 1.9]
SpO_2_ nadir, %	89.0 [84.1 to 94.7]

Abbreviations: 1 min sit to stand (1STS), body mass index (BMI), emergency department (ED), intensive care unit (ICU), length of stay (LOS), oxygen (O_2_), peripheral oxygen saturation (SpO_2_), polymerase chain reaction (PCR), sit to stand (STS). Note: * Data are frequency (percentage) or median [25th to 75th percentile].

## Data Availability

Additional study data can be provided by written request to the corresponding author.
